# Endoscopic endonasal management of giant pediatric craniopharyngioma

**DOI:** 10.3171/2026.7.FOCVID2656

**Published:** 2026-10-01

**Authors:** Michael Z. Cheng, Bastien A. Valencia, Jeeho D. Kim, Michael G. Brandel, John R. Crawford, Jillian Plonsker, Vijay A. Patel, Michael L. Levy

**Affiliations:** 1 Department of Otolaryngology, Rady Children’s Hospital, San Diego;; 2 Department of Head and Neck Surgery, Kaiser Permanente Orange County, Anaheim, California;; 3 Department of Otolaryngology, Mayo Clinic, Rochester, Minnesota;; 4 Department of Otolaryngology, Naval Medical Center San Diego;; 5 Department of Neurosurgery, University of California, San Diego;; 6 Department of Pediatrics, University of California, Irvine;; 7 Department of Neurology, University of California, Irvine; and; 8 Department of Neurosurgery, Rady Children’s Hospital, San Diego, California

**Keywords:** craniopharyngioma, pediatric, endoscopic, skull base surgery

## Abstract

A 13-year-old girl with growth deceleration and multiple endocrinopathies was found to have a giant adamantinomatous craniopharyngioma with hydrocephalus. She underwent an endoscopic endonasal approach with gross-total resection of the tumor with preservation of the hypothalamus. Postoperatively she developed expected panhypopituitarism given pituitary stalk involvement but had no neurological deficits. On 3-year follow-up, there was no tumor recurrence, hypothalamic dysfunction, or hydrocephalus. Pediatric craniopharyngioma remains challenging to manage given complex patterns of spread and proximity to critical neurovascular structures. Advancements in endoscopic techniques have allowed for dynamic visualization and excellent suprasellar access with high cure rates and low morbidity.

The video can be found here: https://stream.cadmore.media/r10.3171/2026.7.FOCVID2656

## Transcript

### 0:20

This surgical video demonstrates an endoscopic endonasal approach for pediatric craniopharyngioma resection.

A 13-year-old girl with normal bone age was evaluated for short stature and poor weight gain. Additional symptoms include intermittent headaches, blurry vision, and hyporexia.

On endocrinologic workup she had low IGF-1 and estradiol, possibly indicative of anterior pituitary dysfunction.

### 0:46

MRI revealed a multilobulated solid and cystic mass measuring up to 4.9 cm. The mass was centered in the posterior aspect of the suprasellar cistern. There was extension and likely obstruction at the level of the third ventricle, resulting in hydrocephalus.

An extra-axial component extended to the right prepontine and cerebellopontine angle cisterns.

The appearance of this tumor with heterogeneity, multiple cystic components, and internal calcifications, combined with the young age of the patient, indicated most likely adamantinomatous craniopharyngioma.

### 1:22

Surgical resection is the standard of care in cases like this one, given the size of the tumor, adamantinomatous type, and hydrocephalus.^1–3^ The extent of resection is debated, but a core principle in the treatment of craniopharyngiomas is to spare the hypothalamus as the consequences of hypothalamic dysfunction are very difficult to treat and have severe impact to quality of life.

Preservation of pituitary function is difficult as the pituitary stalk is often involved by the tumor. However, pituitary dysfunction can be treated with lifelong hormone replacement therapy.

### 1:55

With these in mind, our treatment paradigm is to resect as much of the tumor as is safely feasible in order to minimize the chance of recurrence and need for adjuvant radiation therapy. We avoid manipulation of the hypothalamus and any residual tissue in its vicinity. With this approach, 82% of patients preserved hypothalamic function, and 70% of patients avoided adjuvant radiation in our recently published case series.^4^

### 2:20

We utilize the endoscopic endonasal approach for most craniopharyngiomas. The endoscopic approach is preferred as it provides excellent panoramic visualization of the surgical field and is associated with a lower rate of complications compared to the open approach.^5–7^ We reserve the open orbitozygomatic approach for tumors that have a significant component that is lateral to the carotid artery. For these types of tumors, the open lateral approach allows removal of structures around the vasculature, such as the anterior clinoid and optic strut, to allow for greater maneuverability and dissection.

### 2:54

The endoscopic endonasal approach is associated with some sinonasal morbidity, but this is temporary and most patients return to their baseline 6 months after surgery.^8^ This appears to be the case in pediatric patients as well, with minimal impact on long-term quality of life and craniofacial development.^9^

### 3:11

Our surgery begins with a standard endonasal approach. The middle turbinates are resected and their stumps cauterized for hemostasis. Bilateral sphenoethmoidectomies are carried out using Kerrison rongeurs.

### 3:31

Starting from the sphenoid ostium, we raise our nasoseptal flap, taking care to preserve the superior olfactory strip near the cribriform plate. Given the expected high flow CSF leak from intraventricular dissection of the tumor, we harvest a large flap, extending to the nasal floor for width and the mucocutaneous junction for length.

### 3:57

The flap is elevated in the submucoperichondrial plane, taking care to avoid any tears in the flap. The flap is carefully tucked into the nasopharynx for storage.

### 4:12

Now we perform our posterior septectomy for greater binarial access to the suprasellar region. Despite careful preservation of a dorsal septal strut, there is evidence that nasoseptal flap harvest can result in nasal dorsal deformation in up to 15% of patients. To mitigate this risk, we deploy a reverse septal flap, where the contralateral septal mucosa is incised, flipped around the posterior septum, and inset to the denuded septal cartilage using plain gut sutures.^10^

### 4:50

The sphenoid rostrum is removed to complete binarial access. Sphenoid septations are also removed, and sphenoid sinus mucosa is stripped to prevent mucocele formation and to prepare the site for later mucosal flap coverage.

### 5:22

Bone is removed from the sellar face with a high-speed drill with copious irrigation and Kerrison rongeurs for eggshelling technique. Bone removal extends superiorly to the tuberculum sellae and planum sphenoidale, and laterally toward the optico-carotid recesses. Finally, exposure is extended inferiorly toward the clivus.

### 5:52

The dura is sharply incised. The Ichiban dissector is used to dissect between the capsule of the tumor and the pituitary gland below it.

### 6:19

For the remainder of this dissection, our working corridor through the suprasellar space, between the pituitary gland below and the optic chiasm above.

### 6:32

The tumor is then entered and internally debulked to decompress the tumor, which will provide room for mobilization of the capsule.

### 6:42

With the tumor decompressed, we meticulously dissect the capsule away from critical neurovascular structures in the prepontine space. The attachments here to basilar artery, posterior cerebral arteries, and superior cerebellar arteries are identified and gently lysed. The bilateral oculomotor nerves can be seen here coursing between the PCA and SCA as expected.

### 7:10

With the inferior aspect of the tumor freed, we turn our attention to the intraventricular component. This portion is typically exophytic and without significant attachments. As such, gentle traction in a back-and-forth motion mobilizes the tumor capsule without injury to surrounding structures.

### 7:59

Following tumor removal, a clear view of the third ventricle, foramen of Monro, and choroid plexus is appreciated.

There may be residual tissue in the vicinity of the hypothalamus, but in the interest of preserving hypothalamic function this region is left alone.

### 8:16

The high flow CSF leak resulting from the dissection necessitates a multilayered reconstruction. We also typically insert a lumbar drain to divert CSF in the immediate postoperative period. This also helps decrease pneumocephalus.

### 8:31

For the reconstruction, DuraGen is first placed as an inlay graft using a modified gasket seal technique. The vascularized nasoseptal flap is rotated and overlaid to reline the entire common sphenoid sinus and cover the defect. We then buttress the reconstruction circumferentially with Surgicel, followed by thrombin-soaked Gelfoam in the center of the flap. Two Merocel nasal packs are placed within the nose to ensure gentle pressure along the reconstruction. The anterior septum is held at midline with Doyle splints.

### 9:04

Immediate postoperative MRI demonstrated a well-vascularized nasoseptal flap and gross-total resection of the tumor without residual disease.

### 9:13

Postoperatively, the patient was neurologically intact without any CSF leakage and was discharged after an unremarkable hospitalization.

### 9:24

In the long term, the patient had a good neurological outcome with intact vision. She underwent formal neuropsychological evaluation about a year after surgery and was noted to have normal function in all cognitive and social domains, including attention and concentration. She remains as such at her most recent visits more than 3 years after surgery and is progressing well in school.

### 9:46

She continues to have expected panhypopituitarism and remains on DDAVP, growth hormone, levothyroxine, hydrocortisone, and progesterone and estrogen. However, she has intact hypothalamic function without significant changes in body weight, energy levels, sleep, mood, or behavior.

### 10:06

She remains recurrence-free at her 3-year follow-up. Follow-up neuroimaging more than 3 years postoperatively demonstrates absence of disease recurrence, a well-vascularized nasoseptal flap, and resolution of hydrocephalus.

## Disclosures

The authors report no conflict of interest concerning the materials or methods used in this study or the findings specified in this publication.

## Author Contributions

Primary surgeon: Patel, Levy. Assistant surgeon: Plonsker. Editing and drafting the video and abstract: Patel, Cheng, Kim, Brandel, Crawford, Levy. Critically revising the work: all authors. Reviewed submitted version of the work: Patel, Cheng, Valencia, Kim, Brandel, Crawford, Plonsker. Approved the final version of the work on behalf of all authors: Patel. Supervision: Patel, Crawford, Levy.

## Supplemental Information

### Patient Informed Consent

The necessary patient informed consent was obtained in this study.
